# Causal Modeling to Mitigate Selection Bias and Unmeasured Confounding in Internet-Based Epidemiology of COVID-19: Model Development and Validation

**DOI:** 10.2196/31306

**Published:** 2022-07-21

**Authors:** Nathaniel Stockham, Peter Washington, Brianna Chrisman, Kelley Paskov, Jae-Yoon Jung, Dennis Paul Wall

**Affiliations:** 1 Neurosciences Interdepartmental Program Stanford University Palo Alto, CA United States; 2 Department of Bioengineering Stanford University Stanford, CA United States; 3 Biomedical Informatics Program Stanford University Stanford, CA United States; 4 Department of Biomedical Data Science Stanford University Stanford, CA United States; 5 Department of Pediatrics Stanford University Stanford, CA United States

**Keywords:** selection bias, COVID-19, epidemiology, causality, sensitivity analysis, public health, surveillance, method, epidemiologic research design, model, bias, development, validation, utility, implementation, sensitivity, design, research, epidemiology

## Abstract

**Background:**

Selection bias and unmeasured confounding are fundamental problems in epidemiology that threaten study internal and external validity. These phenomena are particularly dangerous in internet-based public health surveillance, where traditional mitigation and adjustment methods are inapplicable, unavailable, or out of date. Recent theoretical advances in causal modeling can mitigate these threats, but these innovations have not been widely deployed in the epidemiological community.

**Objective:**

The purpose of our paper is to demonstrate the practical utility of causal modeling to both detect unmeasured confounding and selection bias and guide model selection to minimize bias. We implemented this approach in an applied epidemiological study of the COVID-19 cumulative infection rate in the New York City (NYC) spring 2020 epidemic.

**Methods:**

We collected primary data from Qualtrics surveys of Amazon Mechanical Turk (MTurk) crowd workers residing in New Jersey and New York State across 2 sampling periods: April 11-14 and May 8-11, 2020. The surveys queried the subjects on household health status and demographic characteristics. We constructed a set of possible causal models of household infection and survey selection mechanisms and ranked them by compatibility with the collected survey data. The most compatible causal model was then used to estimate the cumulative infection rate in each survey period.

**Results:**

There were 527 and 513 responses collected for the 2 periods, respectively. Response demographics were highly skewed toward a younger age in both survey periods. Despite the extremely strong relationship between age and COVID-19 symptoms, we recovered minimally biased estimates of the cumulative infection rate using only primary data and the most compatible causal model, with a relative bias of +3.8% and –1.9% from the reported cumulative infection rate for the first and second survey periods, respectively.

**Conclusions:**

We successfully recovered accurate estimates of the cumulative infection rate from an internet-based crowdsourced sample despite considerable selection bias and unmeasured confounding in the primary data. This implementation demonstrates how simple applications of structural causal modeling can be effectively used to determine falsifiable model conditions, detect selection bias and confounding factors, and minimize estimate bias through model selection in a novel epidemiological context. As the disease and social dynamics of COVID-19 continue to evolve, public health surveillance protocols must continue to adapt; the emergence of Omicron variants and shift to at-home testing as recent challenges. Rigorous and transparent methods to develop, deploy, and diagnosis adapted surveillance protocols will be critical to their success.

## Introduction

Accurate estimation of disease parameters is a fundamental problem in epidemiology. The internal and external validity of epidemiological studies is threatened by unmeasured confounding and selection bias [1,2]. There is an extensive and sophisticated literature focused on mitigating these threats by study design and poststudy statistical adjustment [3-5]. In particular, the randomization paradigm for treatment assignment and sample selection has served at the de facto standard for identifying causal effects and point estimates of disease parameters in a target population. However, even studies with perfect randomization can still suffer from unmeasured confounding and selection bias via a variety of phenomena, such as participant noncompliance, unit nonresponse, incomplete registers of the target population, and data collection failures [6]. In the past decade, there have been several advances in the theoretical treatment of these threats, particularly in the graphical causal modeling literature, where the problems of selection bias and unmeasured confounding have received a comprehensive theoretical treatment [7-9]. Although these recent methods provide a clear conceptual and mathematical framework, they have yet to be routinely deployed in the epidemiological community at large [10,11].

This gap is particularly acute in internet-based public health and surveillance. Internet-based sampling in general suffers from unknown selection mechanisms on largely unobservable and dynamic populations, making traditional adjustment methods that require external data about the target population vulnerable to model violation. Previous studies that augmented traditional surveillance mechanisms with internet-based data have proved highly successful at imputing missing or time-delayed information [12,13]. However, it is challenging to model emerging pathogens and adapt to changing internet user behavior across time and social context [14]. This fundamental difficulty was demonstrated vividly early in the COVID-19 pandemic as several highly sophisticated crowdsourced internet-based surveillance efforts were launched in response to the pandemic [15-21]. Despite explicit support by global social media and web service vendors, these early efforts yielded significantly biased estimates of key epidemiological parameters [22-26]. Internet-based epidemiology must adopt methodological approaches appropriate to the dynamic and unobservable features of internet populations.

In this work, we seek to address this gap between recent theoretical developments and the current practice of internet-based public health surveillance. We present structural causal modeling as a guide to epidemiological judgement through encoding epidemiological knowledge into models that can be tested using sample data, and we describe a general graphical method for deriving falsifiable model conditions. Importantly, this approach can be deployed using only the sampled data, whereas traditional methods for detecting confounding and selection bias require some information about the unsampled or missing data from units with partial data or external data, such as census or health care system medical records [1]. For novel and dynamic phenomena, the required external information may be unavailable, unreliable, or impractical to collect in the timespan available. Our objective is to demonstrate the practical utility of model diagnosis and selection using statistical criteria derived from structural causal models.

## Methods

### Structural Causal Models

**Figure 1:**
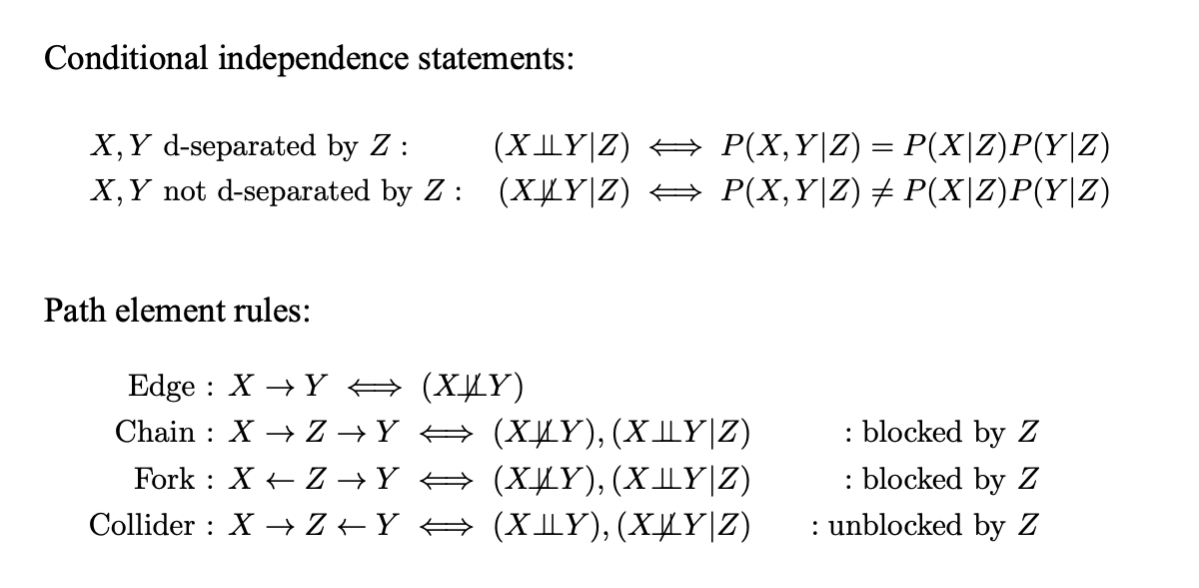
Conditional independence statements and d-separation rules.

Structured causal models permit the formal encoding of causal mechanisms and have been extended to formally analyze studies in the presence of selection bias and unmeasured confounding. The mathematical tool necessary for this work is d-separation on directed acyclic graphs (DAGs). Here, we briefly review d-separation notation and concepts. We can represent a probability distribution as a DAG where nodes represent variables X, Y, and Z and edges represent functional dependencies between variables. The formalism of d-separation is a mapping between the DAG of a probability distribution and the conditional independencies of that distribution; this is stated formally in the conditional independence statements of Figure 1. To state that variables X and Y are d-separated by Z is to state that X and Y are conditionally independent if conditioned on variable Z. Conversely, if X and Y are not d-separated by Z, then X and Y are conditionally dependent if conditioned on Z. A path in a DAG is a sequence of edges (regardless of direction), and every path can be decomposed into a sequence of path elements of edges, chains, forks, and colliders, as shown in Figure 1. The variables X and Y are d-separated in the DAG if all paths from X to Y in the graph are “blocked.” Intuitively, a path from X to Y is blocked if no information about X can be inferred from observing Y via information transferred along that path.

The d-separation path element rules determine whether a path between X and Y is blocked. A path between X and Y can be blocked in 2 different ways by conditioning on a set of variables W. If the path contains a fork or a chain element, then it is blocked if the middle variable (Z in Figure 1) in at least 1 of the fork or chain elements is in W. If the path contains a collider element, then the path is blocked only if the middle variable is not in W. Conditioning on the middle variable of a collider element can unblock the path and make X and Y not d-separated. To illustrate this concept more explicitly, consider 2 independent binary 0,1 variables X and Y, where Z = X + Y. If we condition on Z such that Z=1, then X and Y would appear anticorrelated (nonindependent) because samples with X=Y=0 and X=Y=1 are, by definition, never observed in the subset where Z=1. This effect is known as *collider bias* and is a major source of selection bias in epidemiological studies. For further reading, there are several good introductions to d-separation in graphical models [6,9].

The only additional conceptual step necessary for analyzing selection bias is to add the sampling mechanism to the initial causal graph G to create the augmented causal graph G_s_. The encoded sampling mechanism determines the value of the sampling indicator variable S, where S=1 if the unit was sampled and S=0 otherwise. Additionally, any mechanism that filters data after primary collection induces an additional selection bias and must also be encoded in G_s_. The augmented graph G_s_ obeys d-separation rules, but for clarity, the sampling indicator S node is depicted in G_s_ with a double ring to emphasize that S=1 for all samples by definition; all d-separation statements in G_s_ must be evaluated conditional on S=1.

For any graph G_s_, the s-recoverability condition states that for any variables Y and X in G_s_, the distribution of the sample P(Y|X,S=1) is identical to the distribution of the target population P(Y|X) if and only if Y and S are d-separated by X [8]. Assuming the graph is faithful, S and Y conditioned on X are independent if and only if there is no selection bias or unmeasured confounding. It is not possible to directly test for independence between S and Y using sample data, because is no variation as S=1 by definition, but other surrogate variables in the sample data can be used to test independence of S and Y.

**Figure 2:**
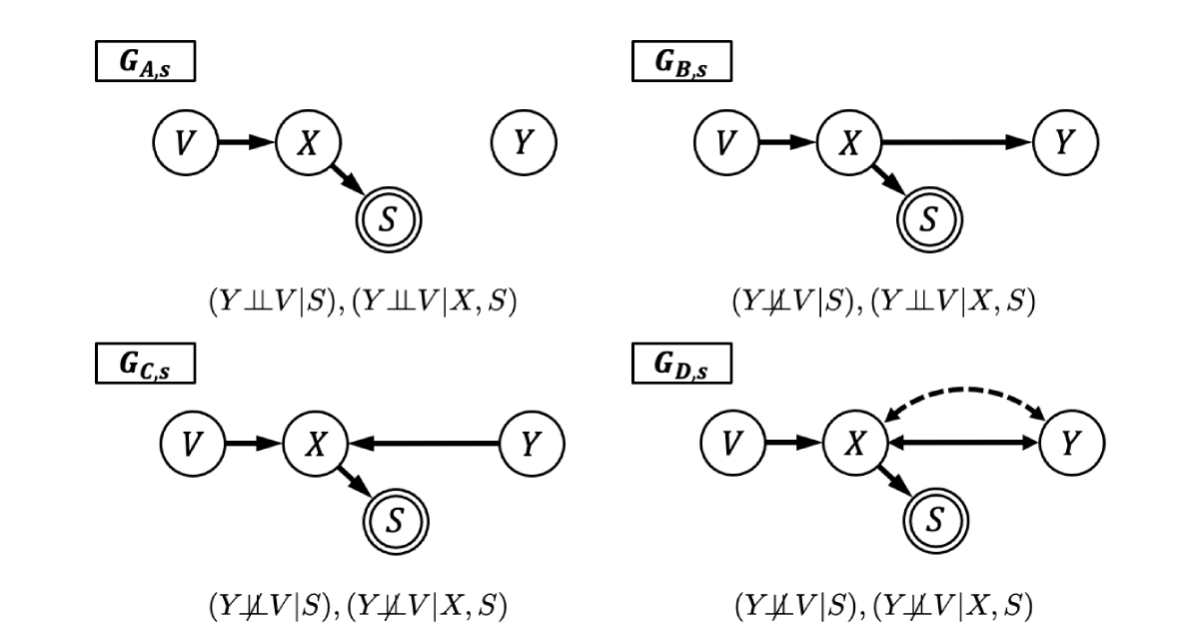
Example causal graphs with selection bias and unmeasured confounding.

We demonstrate this principle in Figure 2 using the causal graphs G_A,s_, G_B,s_, G_C,s_, and G_D,s_ with outcome variable Y, instrumental variable V on variable X relative to Y, and a sample indicator variable S determined by X. Here we define an instrumental variable V on X relative to Y as a variable V that is not independent of X (V and X not d-separated), but V is independent of Y conditional on X (V and Y are d-separated by X). In graph G_A,s_, the variables V and Y are trivially d-separated (d-separated without any blocking variables) and therefore independent. If there is any selection bias (G_B,s_, G_C,s_) or unmeasured confounding (G_D,s_), then V and Y are not trivially d-separable and are not independent. For example, suppose the null hypothesis of statistical independence between V and Y is rejected in the sampled data. Then the graph G_A,s_ is not compatible with the sample data, but alternative graphs G_C,s_, G_B,s_, and G_D,s_ are compatible with nonindependent V and Y and should be considered. Any augmented causal graph G_s_ entails a set of conditional independencies that can be statistically tested using only the sample data. For more complex graphs, there are several software tools that will compute all the entailed independencies, of which *dagitty* is perhaps the most user friendly [26].

In this work, we focus on graphical modeling as a formalism to aid epidemiological judgment. Epidemiological knowledge tightly constrains the set of possible explanatory scenarios for a given context; the difficultly is choosing which of these scenarios is most plausible. Statistically testing the independencies implied by the causal graph encoding is a direct method to select between scenarios. We now demonstrate this approach in an applied problem of estimating the cumulative infection rate CI_P_ of SARS-CoV-2 in the COVID-19 New York City (NYC) spring 2020 epidemic through a prospectively collected crowdsourced internet survey.

### Recruitment

Initial crowdsourced epidemiology efforts in the COVID-19 pandemic focused on surveys collected from a variety of internet sources and target populations. Instead of recruiting via major internet platforms such as Facebook and Google, we recruited our survey participants from the Amazon Mechanical Turk (MTurk) crowdworking platform. MTurk is an internet-based labor market where a research group or business (*requesters*) can create and disseminate a human intelligence task (HIT) to a distributed human labor pool (*workers*) that can accept and complete these tasks for a known monetary reward upon satisfactory completion of the task. A HIT can range from transcribing an audio file to a personality survey, and requesters can restrict the task workers within a specific geographic area or demographic subset. All MTurk workers in the United States are adults of age 18 years or older.

We chose the MTurk population for 2 reasons. First, MTurk has been successfully used by many academic groups, including our own, across a broad array of disciplines [27-32]. Second, the demographics and health status of the MTurk worker population in the United States has been repeatedly characterized and has remained stable through time, closely matching the racial and ethnic composition of the United States but skewing toward women, a younger age, worse mental health, and lower income than the US population [28]. Any MTurk worker registered as residing in New York State or New Jersey was permitted to respond to the survey via the MTurk HIT job posting with restriction that a worker could respond only once per survey period. No other restrictions or invitation mechanisms were used in either survey period.

### Human Subject Research Ethical Statement

This research was not found to be considered human subject research as the survey did not collect any personally identifying information or set of information that could be reidentifying, in compliance with MTurk’s policy prohibiting any transmission of workers’ personally identifiable information to requesters and Stanford University research policy GUI-H12. Research was carried out in a way that followed ethical guidelines set by the Declaration of Helsinki. All MTurk tasks are carefully reviewed before being posted, and MTurk workers are able to accept but then refuse to complete any task or any part of a task at any point in time. Furthermore, the survey task included an introduction page that informed the respondents of the purpose and content of this survey and for what purposes their response data would be used.

### Overview of Survey Design

We collected primary data from the MTurk population listed as currently residing in New Jersey or New York. Data for surveys s_1_ and s_2_ were collected in 2 successive periods: April 11-14 and May 8-11, 2020. During this period, both New York and New Jersey were under a statewide stay-at-home order that greatly restricted travel and prohibited public gatherings [33,34]. We collected primary data from 2 survey periods to estimate the trajectory of the spring 2020 COVID-19 epidemic in NYC and assess the stability of model selection across 2 different phases of the epidemic. The context of NYC in spring 2020 was chosen because it was 1 of the first major COVID-19 epidemics. A Qualtrics survey was created for each survey run, with a reward (median completion time) for the surveys of US $1 (5 minutes) and US $1.25 (6 minutes) for s_1_ and s_2_, respectively. This reward is consistent with other MTurk HITs for the time required. Before accepting the task, the participant was aware of the overall survey subject (COVID-19) and the monetary reward for completion. We excluded responses from participants that were incomplete or out-of-area, as determined by geolocation information provided by Qualtrics. The included responses were split by collection period and aggregated into 3 nested geographic areas: New Jersey and New York (NJ/NY), the section of the New York City Combined Statistical Area contained within New Jersey and New York (NYC CBSA), and NYC proper.

Before answering any questions, the survey asked each participant (respondent) to privately list their 5 closest peer relationships (relations) with whom they typically socialize in person. There was large variation in the number of contacts for each person during the mandatory stay-at-home orders. Instead of asking respondents about their total number of contacts, we asked about their closest peer relationships because these are the set of persons whose current health status and household characteristics would most likely be known to the respondents. Furthermore, we only asked about 5 relations to minimize the time to complete the survey. The survey queried each respondent about the demographic, employment characteristics, and possible COVID-19 symptoms of both themselves and their relations. The survey also queried each respondent about both their household and their relations’ households, including household size and whether any member had a confirmed SARS-CoV-2 infection since March 15, 2020. These questions were chosen to permit comparison of respondents and relations to known census demographic data and to estimate the cumulative number of infected households and individuals within a specified geographic area. The survey material is included in Multimedia Appendices 1 and 2.

### Statistical Analysis

#### Estimator Definition

We defined a household-based cumulative infection rate estimator 
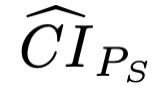
 on a sample P_S_ for the cumulative infection rate as:

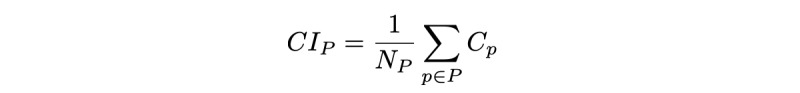



where C_p_ is an indicator variable for the confirmed SARS-CoV-2 infection status of person p in a population P of size N_P_. We defined the household secondary attack rate (SAR_h_) as the ratio of secondary household cases to the total population of exposed household members. We can write SAR_h_ formally as:

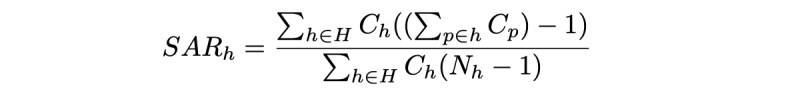



where H is the set of unique households in population P, indicator variable C_h_=1 for if there is at least 1 SARS-CoV-2 infection in household h, and N_h_ the size of household h in H. Let the total population be defined as the sum of the household members N_P_=Σ_hεH_ N_h_. We can then rewrite CI_P_ in terms of households as:

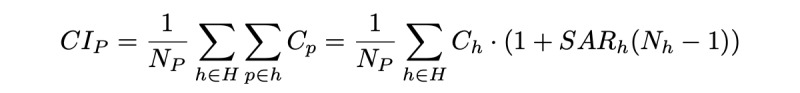



We then defined the estimator 
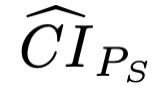
 of CI_P_ on a sample P_S_ as:

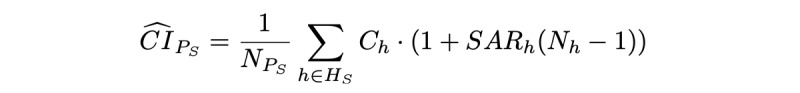



with unique households H_S_. The estimator 
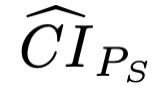
 is consistent as H_S_ goes to H if P(C_h_, N_h_|h in H_s_)=P(C_h_, N_h_) of the target population. In the special case of SAR_h_=0, 
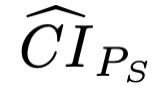
 is an unbiased estimator of the cumulative household infection rate CI_H_ under the less restrictive condition of P(C_h_|S=1)=P(C_h_).

#### Structural Causal Models

**Figure 3:**
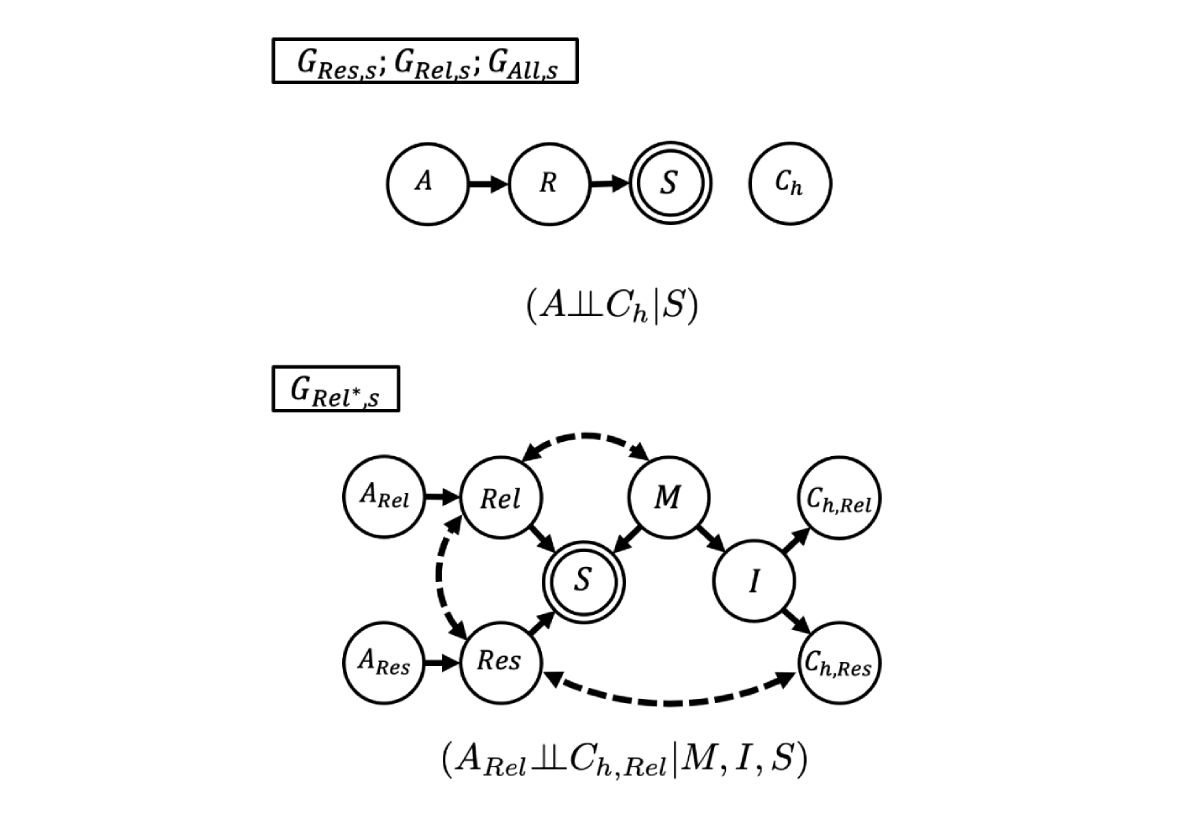
Alternative causal graphs.

The survey data were modeled in a causal graph encoding the variables and assumptions, as depicted in Figure 3. Every person in the population P was assigned 2 indicator variables Res_i_ and Rel_ij_ and the outcome variables C_h,Res,i_ and C_h,Rel,ij_. The variable Res_i_=1 if person p_i_ is a respondent to the survey, and Rel_ij_=1 if p_i_ in P would choose person p_j_ as a relation in the context of this survey. In the sample, the set of respondents is P_S,Res_={p_i_|Res_i_=1}, the set of relations is P_S,Rel_={p_j_|Rel_ij_=1, p_i_ in P_S,Res_}, and the total sample is P_S_=P_S,Res_ ∪ P_S,Rel_. The outcome variable C_h,Res,i_=1 if there is at least 1 confirmed SARS-CoV-2 infection in the household of respondent p_i_ in P_S,Res_, and C_h,Rel,ij_=1 if there is at least 1 confirmed infection in the household of relation p_j_ in P_S,Rel_. For each response, there is 1 respondent and 5 relations chosen by that respondent. In the causal graphs in Figure 3, the subgraph that includes variables pertaining to relations is replicated identically for all 5 relations. Given that this was an anonymous internet survey, we assumed no information bias due to intentional misrepresentations on the part of the respondents or relations. We also assumed there was no information bias due to testing constraints at the level of the household.

We defined 4 possible causal models depicted in Figure 3, augmented with the sampling indicator variable S. All variables were conditioned on a common geographic area, which was suppressed in the graphs and notation for clarity.

The first 3 causal models G_Res,s_, G_Rel,s_, and G_All,s_ all shared the same causal graph, as represented in the first graph in Figure 3, and differed only in terms of the data used. The first causal graph encoded that the age variable A of a person influences whether they respond as a relation/respondent in variable R, which in turn determines whether they are in the sample S. Furthermore, the household infection status C_h_ of the person was assumed to be unrelated to the other variables. The first causal model G_Res,s_ only used data on respondents, whereas the second model G_Rel,s_ only used data on relations. The third model G_All,s_ combined respondents’ and relations’ data.

The second graph in Figure 3 corresponds to the fourth causal model G_Rel*,s_, which encoded a possible confounding between S and C_Res_ and between S and C_Rel_. In particular, G_Rel*,s_ modeled the case where the respondents’ household status C_h,Res_ is related to the relations’ household status C_h,Rel_ through a transmission event I between a respondent and a relation due to the recent close contact event M. The variables I and M d-separated the sampling indicator S and the outcome of interest C_h,Rel_, but the infection event I was unobservable. However, if we filtered the samples so that M=0 (excluding relations with close contact events with the respondent), then I=0 for the retained samples because there can be no transmission without close contact; filtering on M=0 effectively conditioned on M=0 and I=0. Filtering on M also induced another selection bias modeled by the edge M to S, but S and C_h,Rel_ remained d-separated as did C_h,Rel_ and A_Rel_. Therefore, the graph G_Rel*,s_ implied that P(C_h,Rel_|M=0,I=0,S=1)=P(C_h_) and furthermore implied that C_h,Rel_ and A_Rel_ are statistically independent. Effectively, this model excluded information from respondents and excluded relations that had recent in-person contact with the respondents. Practically, this model reduced overestimation of the cumulative infection rate due to possible common causes of the infection status of respondents and relations, such as when a respondent transmits an infection to a relation.

These 4 models can be compared and ranked empirically by statistical tests of the conditional independences implied by their d-separation conditions. Each causal model in Figure 3 has at least 1 independence statement that is testable using the observed data on the age and household infection status of the respondents and relations. From 1 statistical test of the independence statement, we can distinguish which causal model is compatible with the data for each survey period using only the survey sample data. This is an important methodological point, given that the current practice for model diagnosis and selection assumes strong prior knowledge about the target population on several demographic variables. No external data are required for this type of diagnostic analysis, which is a key advantage when the target population is unstudied, inaccessible, or dynamic through time.

We evaluated the causal models by statistically testing the implied independence of A; C_h_ in models G_Res,s_, G_Rel,s_, and G_All,s_; and A_Rel_ and C_h,Rel_ in model G_Rel*,s_ using the Fisher exact test for independence. For each model, we filtered the data, as specified in the model, median-split the age variable, and performed Fisher exact tests on the 2×2 table of the age group (A_0_, A_1_) by house infection status (C_h,0_, C_h,1_) with point test statistics shown in Table 1. Ages for respondents and relations were randomly assigned within the recorded 5-year bin across independent replications (n=1000). The median odds ratio with 95% CIs and median *P* values are reported in Table 1.

**Table 1:** Model selection by conditional independence tests.

Survey		Odds ratio (95% CI)	*P* value	Sample size, N
**G_Res,s_**				
	s_1_	0.802 (0.765-0.922)	.77	527
	s_2_	0.271 (0.202-0.438)	.01	513
**G_Rel,s_**				
	s_1_	0.955 (0.885-1.026)	.90	2635
	s_2_	0.634 (0.581-0.694)	.01	2565
**G_All,s_**				
	s_1_	0.919 (0.824-1.007)	.73	3162
	s_2_	0.572 (0.525-0.614)	<.001	3078
G_Rel*,s_				
	s_1_	0.977 (0.855-1.130)	.99	1340
	s_2_	1.472 (1.216-1.823)	.28	1104

In the general case, there will be no ground truth to compare the model against. However, in this study, we assessed the model performance directly. Due to the particular conditions of the NYC epidemic, the performance of the 
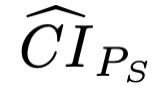
 estimator in each causal model can be evaluated directly from the CI_P_ reported by the New Jersey and New York State health departments. Under the test rationing and home quarantine policies of New York and New Jersey during the spring 2020 epidemic, diagnostic real-time reverse transcription polymerase chain reaction (rRT-PCR) SARS-CoV-2 tests were restricted to individuals hospitalized with COVID-19 symptoms. Households with a member who tested positive were required by law to quarantine [35-40]. Although multiple members of a given household might have SARS-CoV-2 infections, no additional rRT-PCR tests would be performed on other household members unless they were hospitalized. Therefore, the reported CI_P_ is as if SAR_h_=0 where no secondary household cases are reported. We aggregated confirmed SARS-CoV-2 infections as reported by the New York and New Jersey governments for each date and geographic area (NJ/NY, NYC CBSA, NYC) and calculated the CI_P_ relative to the American Community Survey (ACS) population for each. To evaluate the performance of each model, we computed the cumulative infection rate estimator 
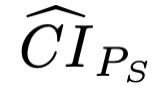
 at SAR_h_=0 and calculated its relative bias from the reported CI_P_ for each area and period.

To demonstrate the practical epidemiological utility of this type of internet-based sampling, we calculated 
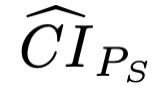
 for different values of SAR_h_ using the causal model most compatible with the primary data, deriving 95% CIs by bootstrap resampling (n=1000).

## Results

### Demographics

In total, 527 and 513 responses met the inclusion criteria from surveys s_1_ and s_2_, respectively. Demographic information is summarized as frequencies for each collection period, with Pearson chi-squared tests performed to compare raw counts to demographic distributions in the 2018 ACS update of the US Census Bureau (Table 2).

Significant age skews were apparent across all survey periods, with both respondents and relations skewing significantly younger than the known population distribution, while sex distributions were not significantly different than the ACS estimate for New York and New Jersey. This large age skew made the sample highly unrepresentative of the target population, but with a correctly specified causal model, it was possible to obtain an unbiased estimate of the cumulative infection rate CI_P_, as we next demonstrate through model diagnosis, selection, and evaluation.

**Table 2:** Demographic characteristics of survey samples.

Characteristics			Respondents, n(%)			Relations, n(%)			Combined, n(%)			ACS^a^ (%)
		Survey s_1_ (N=527)		Survey s_2_ (N=513)	Survey s_1_ (N=2635)		Survey s_2_ (N=2565)	Survey s_1_ (N=3162)		Survey s_2_ (N=3078)		
**Age (years)**												
	<19	2 (0.4)		5 (1.0)	264 (10.0)		356 (13.9)	266 (8.4)		361 (11.7)	22.7	
	19-29	184 (34.9)		192 (37.4)	620 (23.5)		575 (22.4)	804 (25.4)		767 (24.9)	15.2	
	30-39	170 (32.3)		163 (31.8)	531 (20.2)		525 (20.5)	701 (22.2)		688 (22.4)	13.2	
	40-49	89 (16.9)		77 (15)	400 (15.2)		344 (13.4)	489 (15.5)		421 (13.7)	12.9	
	50-59	56 (10.6)		46 (9.0)	367 (13.9)		346 (13.5)	423 (13.4)		392 (12.7)	14.1	
	60-69	19 (3.6)		24 (4.7)	284 (10.8)		294 (11.5)	303 (9.6)		318 (10.3)	11.2	
	≥70	7 (1.3)		6 (1.2)	169 (6.4)		125 (4.9)	176 (5.6)		131 (4.3)	10.6	
	Chi-square (*df*=6)	475		480	457		358	793		672	N/A^b^	
	*P* value	<.001		<.001	<.001		<.001	<.001		<.001	N/A	
**Sex^c^**												
	N/A	1 (0.2)		3 (0.6)	20 (0.8)		77 (3.0)	21 (0.7)		80 (2.6)	N/A	
	Female	267 (50.7)		286 (55.8)	1353 (51.3)		1303 (50.8)	1620 (51.2)		1589 (51.6)	51.4	
	Male	259 (49.1)		224 (43.7)	1262 (47.9)		1185 (46.2)	1521 (48.1)		1409 (45.8)	48.6	
	Chi-square (*df*=1)	0.08		4.50	0.13		0.97	0.046		3.14	N/A	
	*P* value	.77		.03	.71		.32	.83		.07	N/A	
**Occupation (multiple)**												
	Essential worker	153 (30.0)		140 (27.9)	N/A		N/A	N/A		N/A	N/A	
	Food service	31 (5.9)		27 (5.3)	N/A		N/A	N/A		N/A	N/A	
	Health care	66 (12.5)		69 (13.5)	N/A		N/A	N/A		N/A	N/A	
	Work from home	152 (28.8)		183 (35.7)	N/A		N/A	N/A		N/A	N/A	
	Not working	71 (13.5)		86 (16.8)	N/A		N/A	N/A		N/A	N/A	
	Other	231 (43.8)		173 (33.7)	N/A		N/A	N/A		N/A	N/A	

^a^ACS: American Community Survey of the US Census Bureau.

^b^N/A: not applicable.

^c^Sex inferred from the reported gender identity for comparison with the ACS.

### Model Diagnosis, Selection, and Evaluation

In the first survey period, no model could be rejected at nominal α=0.05, but in the second survey period, only model G_Rel*,s_ could not be rejected. The most likely explanation for why the 4 models were more distinguished in the second period is that the cumulative infection rate increased through the course of the epidemic, giving greater power to detect a statistical dependence in a model, even though the total sample size was similar between periods for each model.

### Model Performance

**Figure 4:**
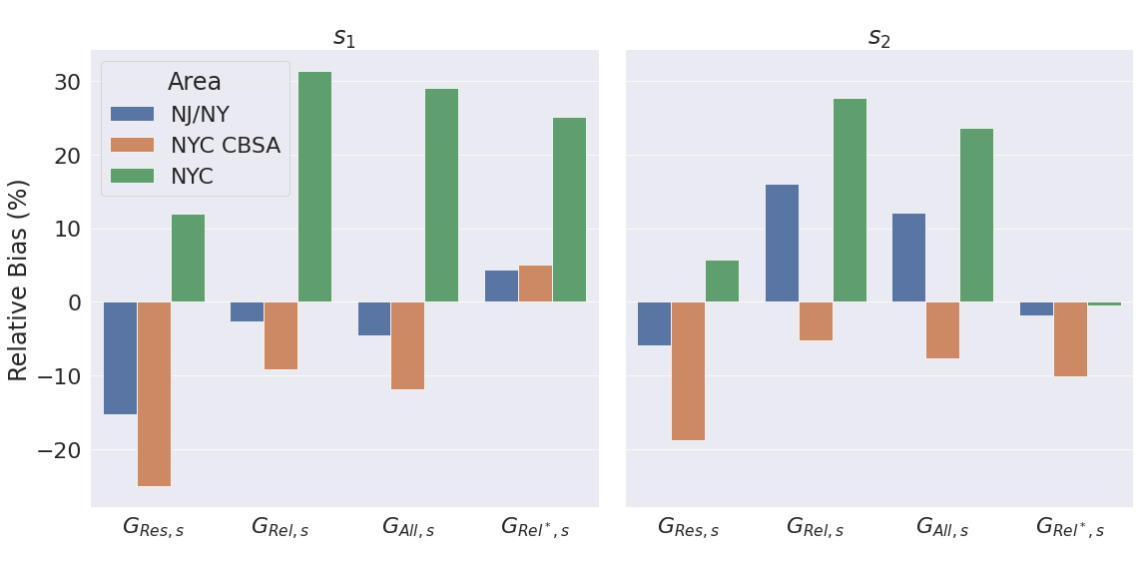
Relative bias of cumulative infection estimates by geographic area, model, and survey period. NJ/NY: New Jersey and New York; NYC: New York City; NYC CBSA: New York City Combined Statistical Area contained within New Jersey and New York.

The model G_Rel*,s_ recovered accurate cumulative infection rate 
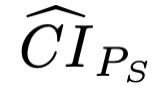
 estimates, with the lowest bias across both survey periods for the full sample (NJ/NY), as displayed in Figure 4, with the estimator variance for all models increasing as the sample size decreased with a smaller geographic area.

### Estimating the Cumulative Infection Rate from the Household Secondary Attack Rate

**Figure 5:**
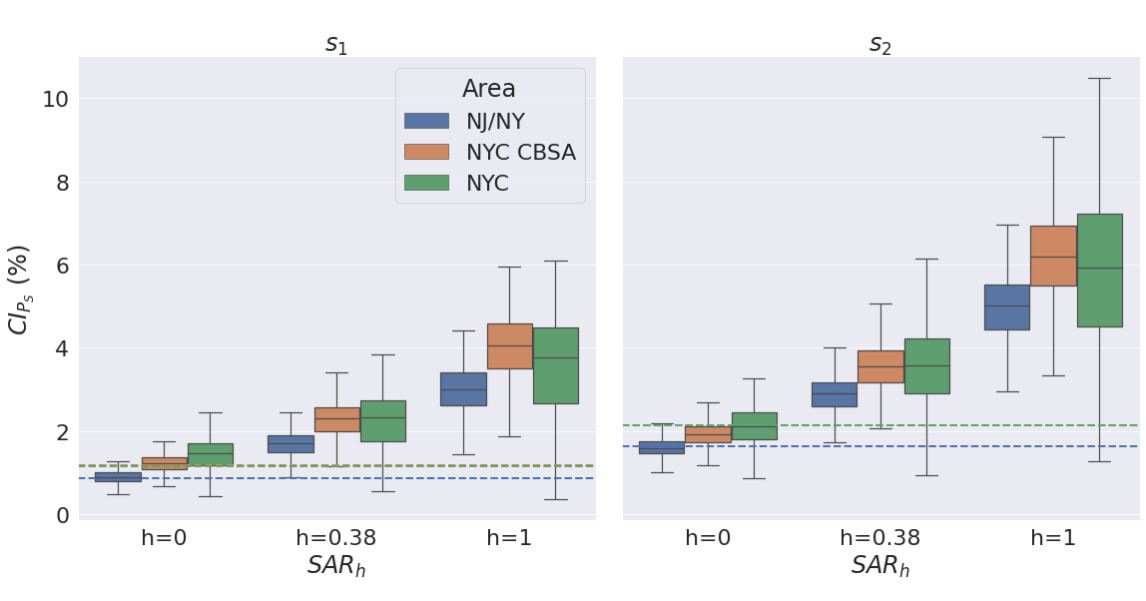
Estimated cumulative infection rate by geographic area, household secondary attack rate (SARh), and survey period. Dashed lines are the reported CI_p_ for the survey period, color-matched to the geographic area. NJ/NY: New Jersey and New York; NYC: New York City; NYC CBSA: New York City Combined Statistical Area contained within New Jersey and New York.

The model G_Rel*,s_ was used to calculate the cumulative infection rate estimator 
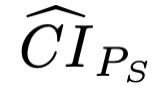
 for different values of SAR_h_, as displayed in Figure 5. For all geographic areas and survey periods, the median 
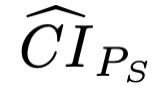
 estimate was approximately 1-4 times higher than the reported cumulative infection rate CI_P_, with upper bounds ranging from 2.5-5 times higher.

### Reported Symptoms Among Respondents and Relations

The number of households with at least 1 confirmed SARS-CoV-2 infection increased by 2 times, and the number of households with at least 1 member recently hospitalized for influenza-like illness (hospital ILI) increased by 1.5 times for both respondents and relations across the 2 survey periods, as shown in Table 3. Despite this, the marginal rates of common symptoms (fever, aches, anosmia, allergy) remained similar across both periods. This highlights the practical difficulties of estimating changes in CI_P_ by using common symptom checklists, as reported by internet surveys.

**Figure 6:**
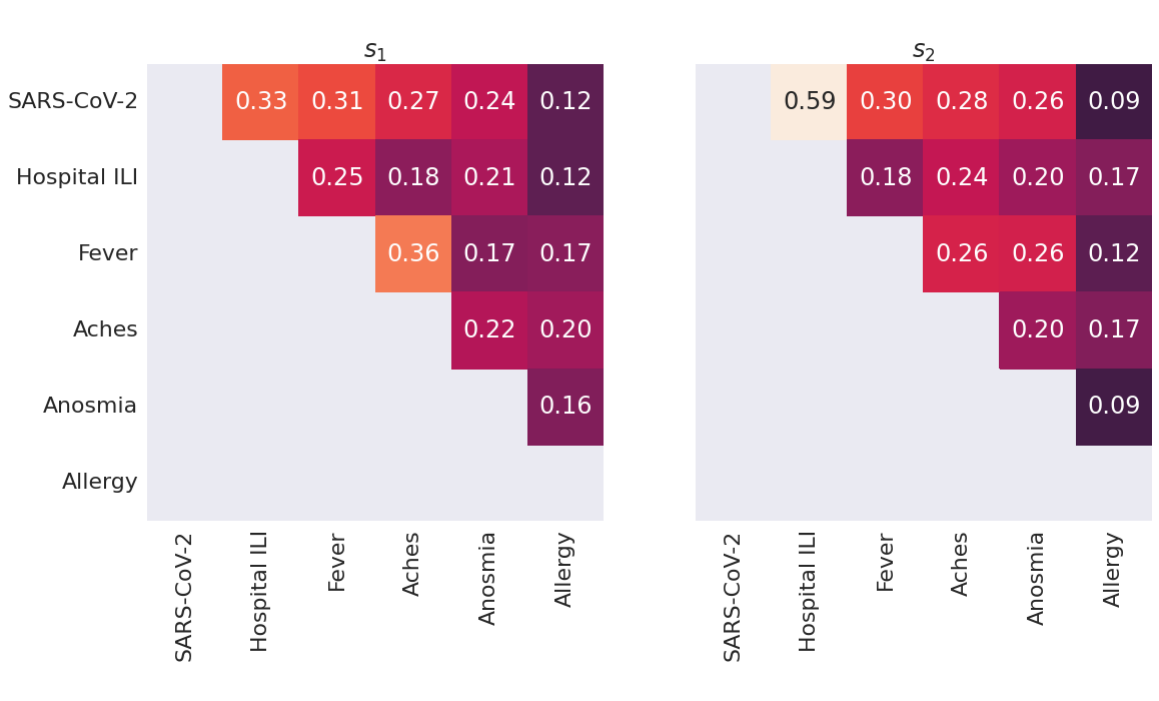
Reported symptom correlations by survey period. SARS-CoV-2 : at least 1 household member tested positive for SARS-CoV-2 infection by rRT-PCR. Hospital ILI: at least 1 household member was recently hospitalized for an ILI. ILI: influenza-like illness; rRT-PCR: real-time reverse transcription polymerase chain reaction.

The correlation between health status indicators and symptoms remained largely the same across both periods (Figure 6). The notable exception is that the correlation between SARS-CoV-2 and hospital ILI increased from the first to the second survey period, presumably corresponding to the conversion from diagnosis to hospitalization as the epidemic progressed.

**Table 3:** Respondent/relation household heath status and symptoms by survey period.

Person		Household health status, n (%)		Reported symptoms, n (%)			
		SARS-CoV-2^a^	Hospital ILI^b,c^	Fever	Aches	Anosmia	Allergy
**Survey s_1_**							
	Respondents (N=1040)	25 (2.4)	24 (2.3)	32 (3.1)	112 (10.8)	45 (4.3)	89 (8.6)
	Relations (N=5200)	155 (3.0)	109 (2.1)	211 (4.1)	325 (6.3)	167 (3.2)	226 (4.4)
**Survey s_2_**							
	Respondents (N=1040)	53 (5.2)	33 (3.2)	23 (2.3)	145 (14.3)	46 (4.5)	104 (10.2)
	Relations (N=5200)	295 (5.8)	169 (3.3)	154 (3.0)	337 (6.6)	180 (3.6)	192 (3.8)

^a^SARS-CoV-2: at least 1 household member had tested positive for SARS-CoV-2 infection by real-time reverse transcription polymerase chain reaction (rRT-PCR).

^b^ILI: influenza-like illness.

^c^Hospital ILI: at least 1 household member was recently hospitalized for an ILI.

## Discussion

### Principle Findings

Using no external data and standard statistical independence tests, we were able to rank and reject all alternative models except the model G_Rel*,s_ that yielded the lowest bias for the cumulative infection rate estimator 
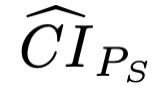
, with a bias of less than 4% on the full sample despite the high skew toward younger ages relative to the target population. Without randomization or representativeness, this study recovered accurate estimates of a key epidemiological parameter using an internet-based crowdsourced population in a dynamic public health crisis using only a few hundred samples.

Although we primarily intend this work to demonstrate the broad utility of graphical models as an aid to epidemiologists, it is worth noting how useful internet-based epidemiology could prove in future epidemics by inspecting the estimates of 
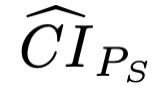
 for varying SAR_h_ (Figure 5). A major source of confusion in the early COVID-19 pandemic was diverging estimates of the cumulative infection rate. By July 27, 2020, there were 228,679 rRT-PCR cumulative confirmed infections in NYC for a reported CI_P_ of 2.65%. However, the actual CI_P_ was estimated to be 23.3% by seroprevalence studies in the July 27-August 13, 2020, period—8.8 times higher [41-44]. A similar difference would imply that the reported CI_P_ of 2.14% by May 8, 2020, corresponded to an actual CI_P_ of ~19%. Using the simple method of estimating 
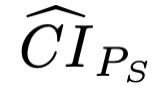
 by SAR_h_=0.38, it would yield intermediate estimates implying upper bounds on the actual CI_P_ of 2.5 and 5 times the reported cumulative infection rate CI_P_.

### Limitations

The limitations of this approach are encoded directly in the set of causal graphs and entail explicit conditions where statistical tests will fail to reject incorrect models. For example, if age is a poor instrumental variable for response status, then with finite data, none of the models may be rejected by statistical tests. In contrast, if age is strongly related to the outcome variable household status C_h_, but not response status, then all models could be rejected, even if there was no selection bias on C_h_. More generally, if there is any relationship between a set of variables, there will be a statistically significant correlation, given sufficient data; therefore, any causal model regardless of its utility will be rejected if statistical tests are applied naively.

These inherent limitations are why we emphasize structural causal models as an aid and not a substitute for epidemiological judgment. The utility of causal modeling is the formal comparison and communication of alternative explanations of the sampled data. For example, in this study, we chose to not model information bias, instead focusing on detecting selection bias. The choice to ignore information bias is explicit in the presented causal graphs; none of them have a subgraph that models an information bias mechanism, such as rRT-PCR test constraints or inaccurate self-reporting. These causal models were constructed with these assumptions for the context and objectives of this study, and similar assumptions may not be acceptable for a different context or objective. The key point is that all these assumptions are made apparent on inspection of the causal graphs.

### Conclusion

The COVID-19 pandemic is an unprecedented event, pushing the limits of the health care system worldwide. Reducing transmission via nonpharmacological interventions has been effective but requires near-real-time and accurate information across all segments of society, information that has been difficult to reliably ascertain. Given the vast divergence of cumulative infection rate estimates across early studies [43] and the consequences for undermining public trust, there is a clear use case for internet-based public health surveillance to rapidly estimate key epidemiological parameters. A major use of internet-based surveys in the COVID-19 pandemic has been estimating the rate of vaccine uptake. The Census Household Pulse and Delphi-Facebook overestimated COVID-19 vaccine uptake to May 2021 by 14% and 17%, respectively, in the United States [44], while a much smaller Axios-Ipsos online survey of a different design overestimated uptake by only 5% in the United States. Internet-based surveys are an important tool with several uses for managing a pandemic, but current methodology is hampered by an inability to successfully detect and mitigate estimate bias. However, looking beyond vaccine uptake, near-term public health interventions, and advances in treatments, COVID-19 continues to evolve along with human societies. Surveillance systems and statistical models that assume centralized reporting may not be as useful with the mass adoption of at-home tests for COVID-19; alternative approaches, such as the social network polling design used in this work, may need to be deployed. For these reasons, we hope that these recent advances in causal modeling theory are adopted by the epidemiological community for current and future epidemics.

## Appendix

Multimedia Appendix 1 Survey materials for survey period 1.

Multimedia Appendix 2 Survey materials for survey period 2.

## Abbreviations

ACS: American Community Survey
DAG: directed acyclic graph
HIT: human intelligence task
ILI: influenza-like illness
MTurk: Mechanical Turk
NJ/NY: New Jersey and New York
NYC: New York City
NYC CBSA: New York City Combined Statistical Area contained within New Jersey and New York
rRT-PCR: real-time reverse transcription polymerase chain reaction

## Data Availability

The data underlying this paper will be shared upon reasonable request to the corresponding author.
