# Supplementary material for: Causal Modeling to Mitigate Selection Bias and Unmeasured Confounding in Internet-Based Epidemiology of COVID-19: Model Development and Validation
Source: JMIR Public Health Surveill. 2022 Jul 21;8(7):e31306. doi: 10.2196/31306 (PMC9307267; doi:10.2196/31306)
Supplement: Multimedia Appendix 1 [file publichealth_v8i7e31306_app1.pdf]

block:p0

Hello there! In response to the spread of Coronavirus we are conducting this survey to monitor and studying how respiratory illnesses transmit through social networks. By completing this survey you are contributing to our current situational awareness of Coronavirus as well as our understanding of respiratory illnesses in general.

Note: We are focusing on New York and New Jersey right now. Please only take this survey if you are currently living in those states, otherwise we will have to reject your response. Thank you.

In this survey we are going to ask you some simple questions about yourself and your close relationships. These questions parse apart how different social, environmental, and genetic risk factors contribute to the spread of respiratory infections. This survey should take between 5 and 10 minutes to complete.

Instruction: Before continuing, could you please take a moment and think of five close relationships. These relationships can be family or friends, but should be people that you know well, communicate with relatively often, and normally socialize with in person. Now, please write down the first name of each of those people, in any order. We can't see it, but writing down the names is important so that you don't lose track of who you're thinking about while taking the survey.

Done that? Thank you!

Now let's start the survey with some warmup questions.

Usually, about how many left handed people (excluding yourself) do you see in person in a week? (Take your best guess)

- 0
- 0 to 1
- 1 to 2
- 2 to 3
- 3 to 4
- 4+

How many left handed people (excluding yourself) did you see this last week?

- 0
- 1
- 2
- 3
- 4
- 5+

Now we're going to ask you a few questions about yourself.

What is your age? (In years)

What is your gender?

- woman
- man
- non-binary
- other

How many siblings do you have?

Are you left or right handed?

- left
- right
- neither/unsure

Are you classified as an essential worker?

- yes
- no
- unsure

What is your usual occupational setting? (Please check all that apply)

- ☐ food service
- ☐ healthcare
- ☐ building maintenance/cleaning
- ☐ working from home
- ☐ not working
- ☐ other

Have you developed any of these symptoms in the last 2 weeks? (Please check all that apply)

- ☐ fever above 101F for multiple days
- ☐ sudden loss of smell
- ☐ persistent and unusual head and body aches
- ☐ unexplained severe allergy symptoms
- ☐ respiratory symptoms requiring medical attention

Including yourself, how many people are currently living in your home?

[ ]

Have you, or anyone living with you, tested positive for the **Flu** in the last 2 weeks?

- ○ yes
- ○ no/unsure

Have you, or anyone living with you, tested positive for **Coronavirus** in the last 2 weeks?

- yes
- no/unsure

Have you, or anyone living with you, been hospitalized for a **new** respiratory illness in the last 2 weeks?

- yes
- no/unsure

Compared to usual, about how many people have you seen in person this last week?

- more than usual
- about the same as usual
- 3/4 of usual
- 1/2 of usual
- 1/4 of usual
- 1/8 of usual
- <1/8 of usual

(Optional) What is your current zipcode? This information very much helps quickly recognizing areas of outbreaks.

Thank you for telling us about yourself. Now we're going to ask you a few

questions about each of the people on the list you wrote down (see instructions at the top if you did not).

Ready? Start by clicking the Next button.

block:p1

Now please consider the **first** person on the list.

Are they your sibling, parent, or child?

- yes
- no

What is their relationship to you?

- sibling
- parent
- child

What type of sibling?

- step sibling
- half sibling; same mother
- half sibling; same father
- full sibling; same mother and father
- twin; identical
- twin; not identical

What type of parent?

- step
- biological

What type of child?

- step
- biological

What is their age? (In years)

What is their gender?

- woman
- man
- non-binary
- other

Are they left or right handed?

- left
- right
- neither/unsure

Where do you usually see them in person? (Please check all that apply)

- ☐ your current residence
- ☐ work
- ☐ commute
- ☐ social events

Have you seen them in person in the last 2 weeks?

- ○ yes
- ○ no

Have they developed any of these symptoms in the last 3 weeks? (Please check all that apply)

- ☐ fever above 101F for multiple days
- ☐ sudden loss of smell
- ☐ persistent and unusual head and body aches
- ☐ unexplained severe allergy symptoms
- ☐ respiratory symptoms requiring medical attention

Including them, about how many people are currently living in their home?

[ ]

Have they, or anyone living with them, tested positive for the **Flu** in the last 2 weeks?

- yes
- no/unsure

Have they, or anyone living with them, tested positive for **Coronavirus** in the last 2 weeks?

- yes
- no/unsure

Have they, or anyone living with them, been hospitalized for a **new** respiratory illness in the last 2 weeks?

- yes
- no/unsure

Thank you for telling us about your first person!

Next, a few questions about the **second** person on your list.

block:p2

Now please consider the **second** person on the list.

Are they your sibling, parent, or child?

- yes
- no

What is their relationship to you?

- sibling
- parent
- child

What type of sibling?

- step
- half sibling; same mother
- half sibling; same father
- full sibling; same mother and father
- twin; identical
- twin; not identical

What type of parent?

- step
- biological

What type of child?

- ◯ step
- ◯ biological

What is their age? (In years)

[ ]

What is their gender?

- ◯ woman
- ◯ man
- ◯ non-binary
- ◯ other

Are they left or right handed?

- ◯ left
- ◯ right
- ◯ neither/unsure

Where do you usually see them in person? (Please check all that apply)

- ☐ your current residence
- ☐ work
- ☐ commute
- ☐ social events

Have you seen them in person in the last 2 weeks?

- ○ yes
- ○ no

Have they developed any of these symptoms in the last 2 weeks? (Please check all that apply)

- ☐ fever above 101F for multiple days
- ☐ sudden loss of smell
- ☐ persistent and unusual head and body aches
- ☐ unexplained severe allergy symptoms
- ☐ respiratory symptoms requiring medical attention

Including them, about how many people are currently living in their home?

[ ]

Have they, or anyone living with them, tested positive for the **Flu** in the last 2 weeks?

- ○ yes
- ○ no/unsure

Have they, or anyone living with them, tested positive for **Coronavirus** in the last 2 weeks?

- yes
- no/unsure

Have they, or anyone living with them, been hospitalized for a **new** respiratory illness in the last 2 weeks?

- yes
- no/unsure

Thank you for telling us about your second person!

Next, a few questions about the **third** person on your list.

block:p3

Now please consider the **third** person on the list.

Are they your sibling, parent, or child?

- yes
- no

What is their relationship to you?

- ◯ sibling
- ◯ parent
- ◯ child

What type of sibling?

- ◯ step
- ◯ half sibling; same mother
- ◯ half sibling; same father
- ◯ full sibling; same mother and father
- ◯ twin; identical
- ◯ twin; not identical

What type of parent?

- ◯ step
- ◯ biological

What type of child?

- ◯ step
- ◯ biological

What is their age? (In years)

[ ]

What is their gender?

- ◯ woman
- ◯ man
- ◯ non-binary
- ◯ other

Are they left or right handed?

- ◯ left
- ◯ right
- ◯ neither/unsure

Where do you usually see them in person? (Please check all that apply)

- ☐ your current residence
- ☐ work
- ☐ commute
- ☐ social events

Have you seen them in person in the last 2 weeks?

- ◯ yes
- ◯ no

Have they developed any of these symptoms in the last 2 weeks? (Please check all that apply)

- ☐ fever above 101F for multiple days
- ☐ sudden loss of smell
- ☐ persistent and unusual head and body aches
- ☐ unexplained severe allergy symptoms
- ☐ respiratory symptoms requiring medical attention

Including them, about how many people are currently living in their home?

[ ]

Have they, or anyone living with them, tested positive for the **Flu** in the last 2 weeks?

- ○ yes
- ○ no/unsure

Have they, or anyone living with them, tested positive for **Coronavirus** in the last 2 weeks?

- ○ yes
- ○ no/unsure

Have they, or anyone living with them, been hospitalized for a **new** respiratory illness in the last 2 weeks?

- yes
- no/unsure

Thank you for telling us about your third person!

Next, a few questions about the **fourth** person on your list.

block:p4

Now please consider the **fourth** person on the list.

Are they your sibling, parent, or child?

- yes
- no

What is their relationship to you?

- sibling
- parent
- child

What type of sibling?

- step
- half sibling; same mother
- half sibling; same father
- full sibling; same mother and father
- twin; identical
- twin; not identical

What type of parent?

- step
- biological

What type of child?

- step
- biological

What is their age? (In years)

What is their gender?

- ◯ woman
- ◯ man
- ◯ non-binary
- ◯ other

Are they left or right handed?

- ◯ left
- ◯ right
- ◯ neither/unsure

Where do you usually see them in person? (Please check all that apply)

- ☐ your current residence
- ☐ work
- ☐ commute
- ☐ social events

Have you seen them in person in the last 2 weeks?

- ◯ yes
- ◯ no

Have they developed any of these symptoms in the last 2 weeks? (Please check all that apply)

- ☐ fever above 101F for multiple days
- ☐ sudden loss of smell
- ☐ persistent and unusual head and body aches
- ☐ unexplained severe allergy symptoms
- ☐ respiratory symptoms requiring medical attention

Including them, about how many people are currently living in their home?

[ ]

Have they, or anyone living with them, tested positive for the **Flu** in the last 2 weeks?

- ○ yes
- ○ no/unsure

Have they, or anyone living with they, tested positive for **Coronavirus** in the last 2 weeks?

- ○ yes
- ○ no/unsure

Have they, or anyone living with them, been hospitalized for a **new** respiratory illness in the last 2 weeks?

- yes
- no/unsure

Thank you for telling us about your fourth person!

Next, a few questions about the **fifth** and final person on your list!

block:p5

Now please consider the **fifth** and final person on the list.

Are they your sibling, parent, or child?

- yes
- no

What is their relationship to you?

- sibling
- parent
- child

What type of sibling?

- step
- half sibling; same mother
- half sibling; same father
- full sibling; same mother and father
- twin; identical
- twin; not identical

What type of parent?

- step
- biological

What type of child?

- step
- biological

What is their age? (In years)

What is their gender?

- ◯ woman
- ◯ man
- ◯ non-binary
- ◯ other

Are they left or right handed?

- ◯ left
- ◯ right
- ◯ neither/unsure

Where do you usually see them in person? (Please check all that apply)

- ☐ your current residence
- ☐ work
- ☐ commute
- ☐ social events

Have you seen them in person in the last 2 weeks?

- ◯ yes
- ◯ no

Have they developed any of these symptoms in the last 2 weeks? (Please check all that apply)

- ☐ fever above 101F for multiple days
- ☐ sudden loss of smell
- ☐ persistent and unusual head and body aches
- ☐ unexplained severe allergy symptoms
- ☐ respiratory symptoms requiring medical attention

Including them, about how many people are currently living in their home?

Have they, or anyone living with them, tested positive for the **Flu** in the last 2 weeks?

- ○ yes
- ○ no/unsure

Have they, or anyone living with they, tested positive for **Coronavirus** in the last 2 weeks?

- ○ yes
- ○ no/unsure

Have they, or anyone living with them, been hospitalized for a **new** respiratory illness in the last 2 weeks?

- ◯ yes
- ◯ no/unsure

Thank you so much for helping us understand how Coronavirus and other respiratory illnesses move through our social lives.

To finish and submit the survey, click the Next button and then the Submit button on the next page.

Thank you again for your time!

Powered by Qualtrics
