# Supplementary material for: Causal Modeling to Mitigate Selection Bias and Unmeasured Confounding in Internet-Based Epidemiology of COVID-19: Model Development and Validation
Source: JMIR Public Health Surveill. 2022 Jul 21;8(7):e31306. doi: 10.2196/31306 (PMC9307267; doi:10.2196/31306)
Supplement: Multimedia Appendix 2 [file publichealth_v8i7e31306_app2.pdf]

block:p0

Note: We are focusing on New York and New Jersey right now. Please only take this survey if you are currently living in those states, otherwise we cannot use your response. Thank you!

REALLY IMPORTANT INSTRUCTION: Before continuing, could you please take a moment and think of five close relationships. These relationships can be family or friends, but should be people that you know well, communicate with relatively often, and normally socialize with in person. Now, please write down the first name of each of those five people into a list. We can't see it, but writing down the names is important so that you don't lose track of who you're thinking about while taking the survey.

Done that? Thank you!

Hello there! In response to the spread of Coronavirus we are conducting this survey to monitor and study how respiratory illnesses transmit through social networks. By completing this survey you are contributing to our situational awareness and understanding of Coronavirus, so thank you for your help!

In this survey we are going to ask you some simple questions about your and your close relationships' social environment. These questions monitor Coronavirus outbreaks and parse apart how different social, environmental, age, and genetic risk factors prevent or encourage infections. This survey should take between 5 and 10 minutes to complete.

Now let's start the survey with some warmup questions.

Before March 2020, about how many left handed people (excluding yourself) did you usually see in person in a week? (Take your best guess)

- 0
- 0 to 1
- 1 to 2
- 2 to 3
- 3 to 4
- 4+

How many left handed people (excluding yourself) did you see this last week?

- 0
- 1
- 2
- 3
- 4
- 5+

Now we're going to ask you a few questions about yourself.

What is your age? (In years)

What is your gender?

- woman
- man
- non-binary
- other

How many siblings do you have?

Are you left or right handed?

- left
- right
- neither/unsure

Are you classified as an essential worker?

- yes
- no
- unsure

What is your usual occupational setting? (Please check all that apply)

- ☐ food service
- ☐ healthcare
- ☐ building maintenance/cleaning
- ☐ working from home
- ☐ not working
- ☐ other

Have you developed any of these symptoms in the last 2 weeks? (Please check all that apply)

- ☐ fever above 101F for multiple days
- ☐ sudden loss of smell
- ☐ persistent and unusual head and body aches
- ☐ unexplained severe allergy symptoms
- ☐ respiratory symptoms requiring medical attention

How many people share your living space? (Including yourself)

How many of those are younger than 19 years?

How many of those are older than 64 years?

Have you, or anyone living with you, tested positive for the **Flu** since January 1st, 2020?

- yes
- no/unsure

Have you, or anyone living with you, ever tested positive for **Coronavirus**?

- yes
- no/unsure

Have you, or anyone living with you, been hospitalized for a **new** respiratory illness since March 15th, 2020?

- yes
- no/unsure

Compared to this time last year, about how many people have you seen in person this last week?

- more than usual
- about the same as usual
- 3/4 of usual
- 1/2 of usual
- 1/4 of usual
- 1/8 of usual
- <1/8 of usual

(Optional) What is your current zipcode? This information very much helps to quickly pinpoint outbreak areas.

Thank you for telling us about yourself. Now we're going to ask you a few questions about each of the people on the list you wrote down (see instructions at the top if you did not).

Ready? Start by clicking the Next button.

block:live_with

Respiratory viruses are often passed between people that are living together because of the long time they spend near each other. To understand how Coronavirus spreads, we need to know who is sharing living space with who.

Remember that nice list of five close relationships you already wrote down? We're going to ask about those 5 people, and whether or not they share living spaces. It doesn't matter if they all live together, all separate, or something in between. We're just need to know who is sharing the same physical environment a lot of the time.

Clarification: For this survey, "living with" **only** means "regularly spending multiple nights a week in same sleeping place".

Ready? Lets start!

Who have **you** been living with on your list since March 20th, 2020? (Please check all that apply)

- ☐ first person on your list
- ☐ second person on your list
- ☐ third person on your list
- ☐ fourth person on your list
- ☐ fifth person on your list
- ☐ none of them

Who has the **first** person on your list been living with since March 20th, 2020? (Please check all that apply)

- ☐ you
- ☐ second person on your list
- ☐ third person on your list
- ☐ fourth person on your list
- ☐ fifth person on your list
- ☐ none of them

Who has the **second** person on your list been living with since March 20th, 2020? (Please check all that apply)

- ☐ first person on your list
- ☐ you
- ☐ third person on your list
- ☐ fourth person on your list
- ☐ fifth person on your list
- ☐ none of them

Who has the **third** person on your list been living with since March 20th, 2020? (Please check all that apply)

- ☐ first person on your list
- ☐ second person on your list
- ☐ you
- ☐ fourth person on your list
- ☐ fifth person on your list
- ☐ none of them

Who has the **fourth** person on your list been living with since March 20th, 2020? (Please check all that apply)

- ☐ first person on your list
- ☐ second person on your list
- ☐ third person on your list
- ☐ you
- ☐ fifth person on your list
- ☐ none of them

Who has the **fifth** person on your list been living with since March 20th, 2020? (Please check all that apply)

- ☐ first person on your list
- ☐ second person on your list
- ☐ third person on your list
- ☐ fourth person on your list
- ☐ you
- ☐ none of them

Thank you for giving us an idea of who has been around who.

Now, we're going to ask you a few questions about each person, to fill in a little detail about them regarding coronavirus.

block:p1

Now please consider the **first** person on the list.

Are they your sibling, parent, or child?

- ○ yes
- ○ no

What is their relationship to you?

- sibling
- parent
- child

What type of sibling?

- step sibling
- half sibling; same mother
- half sibling; same father
- full sibling; same mother and father
- twin; identical
- twin; not identical

What type of parent?

- step
- biological

What type of child?

- step
- biological

What is their age? (In years)

What is their gender?

- ◯ woman
- ◯ man
- ◯ non-binary
- ◯ other

Are they left or right handed?

- ◯ left
- ◯ right
- ◯ neither/unsure

Where do you usually see them in person? (Please check all that apply)

- ☐ your current residence
- ☐ work
- ☐ commute
- ☐ social events

Have you seen them in person in the last 2 weeks?

- ◯ yes
- ◯ no

Have they developed any of these symptoms in the last 2 weeks? (Please check all that apply)

- ☐ fever above 101F for multiple days
- ☐ sudden loss of smell
- ☐ persistent and unusual head and body aches
- ☐ unexplained severe allergy symptoms
- ☐ respiratory symptoms requiring medical attention

Including them, about how many people share their living space?

How many of those are younger than 19 years?

How many of those are older than 64 years?

Have they, or anyone living with them, tested positive for the **Flu** since January 1st, 2020?

- ◯ yes
- ◯ no/unsure

Have they, or anyone living with them, ever tested positive for **Coronavirus**?

- yes
- no/unsure

Have they, or anyone living with them, been hospitalized for a **new** respiratory illness since March 15th, 2020?

- yes
- no/unsure

Thank you for telling us about your first person!

Next, a few questions about the **second** person on your list.

block:p2

Now please consider the **second** person on the list.

Are they your sibling, parent, or child?

- yes
- no

What is their relationship to you?

- sibling
- parent
- child

What type of sibling?

- step
- half sibling; same mother
- half sibling; same father
- full sibling; same mother and father
- twin; identical
- twin; not identical

What type of parent?

- step
- biological

What type of child?

- step
- biological

What is their age? (In years)

What is their gender?

- ◯ woman
- ◯ man
- ◯ non-binary
- ◯ other

Are they left or right handed?

- ◯ left
- ◯ right
- ◯ neither/unsure

Where do you usually see them in person? (Please check all that apply)

- ☐ your current residence
- ☐ work
- ☐ commute
- ☐ social events

Have you seen them in person in the last 2 weeks?

- ◯ yes
- ◯ no

Have they developed any of these symptoms in the last 2 weeks? (Please check all that apply)

- ☐ fever above 101F for multiple days
- ☐ sudden loss of smell
- ☐ persistent and unusual head and body aches
- ☐ unexplained severe allergy symptoms
- ☐ respiratory symptoms requiring medical attention

Including them, about how many people share their living space?

How many of those are younger than 19 years?

How many of those are older than 64 years?

Have they, or anyone living with them, tested positive for the **Flu** since January 1st, 2020?

- ○ yes
- ○ no/unsure

Have they, or anyone living with them, ever tested positive for **Coronavirus**?

- yes
- no/unsure

Have they, or anyone living with them, been hospitalized for a **new** respiratory illness since March 15th, 2020?

- yes
- no/unsure

Thank you for telling us about your second person!

Next, a few questions about the **third** person on your list.

block:p3

Now please consider the **third** person on the list.

Are they your sibling, parent, or child?

- yes
- no

What is their relationship to you?

- sibling
- parent
- child

What type of sibling?

- step
- half sibling; same mother
- half sibling; same father
- full sibling; same mother and father
- twin; identical
- twin; not identical

What type of parent?

- step
- biological

What type of child?

- step
- biological

What is their age? (In years)

What is their gender?

- ◯ woman
- ◯ man
- ◯ non-binary
- ◯ other

Are they left or right handed?

- ◯ left
- ◯ right
- ◯ neither/unsure

Where do you usually see them in person? (Please check all that apply)

- ☐ your current residence
- ☐ work
- ☐ commute
- ☐ social events

Have you seen them in person in the last 2 weeks?

- ◯ yes
- ◯ no

Have they developed any of these symptoms in the last 2 weeks? (Please check all that apply)

- ☐ fever above 101F for multiple days
- ☐ sudden loss of smell
- ☐ persistent and unusual head and body aches
- ☐ unexplained severe allergy symptoms
- ☐ respiratory symptoms requiring medical attention

Including them, about how many people share their living space?

How many of those are younger than 19 years?

How many of those are older than 64 years?

Have they, or anyone living with them, tested positive for the **Flu** since January 1st, 2020?

- ◯ yes
- ◯ no/unsure

Have they, or anyone living with them, ever tested positive for **Coronavirus**?

- yes
- no/unsure

Have they, or anyone living with them, been hospitalized for a **new** respiratory illness since March 15th, 2020?

- yes
- no/unsure

Thank you for telling us about your third person!

Next, a few questions about the **fourth** person on your list.

block:p4

Now please consider the **fourth** person on the list.

Are they your sibling, parent, or child?

- yes
- no

What is their relationship to you?

- ◯ sibling
- ◯ parent
- ◯ child

What type of sibling?

- ◯ step
- ◯ half sibling; same mother
- ◯ half sibling; same father
- ◯ full sibling; same mother and father
- ◯ twin; identical
- ◯ twin; not identical

What type of parent?

- ◯ step
- ◯ biological

What type of child?

- ◯ step
- ◯ biological

What is their age? (In years)

[ ]

What is their gender?

- ○ woman
- ○ man
- ○ non-binary
- ○ other

Are they left or right handed?

- ○ left
- ○ right
- ○ neither/unsure

Where do you usually see them in person? (Please check all that apply)

- ☐ your current residence
- ☐ work
- ☐ commute
- ☐ social events

Have you seen them in person in the last 2 weeks?

- ○ yes
- ○ no

Have they developed any of these symptoms in the last 2 weeks? (Please check all that apply)

- ☐ fever above 101F for multiple days
- ☐ sudden loss of smell
- ☐ persistent and unusual head and body aches
- ☐ unexplained severe allergy symptoms
- ☐ respiratory symptoms requiring medical attention

Including them, about how many people share their living space?

How many of those are younger than 19 years?

How many of those are older than 64 years?

Have they, or anyone living with them, tested positive for the **Flu** since January 1st, 2020?

- ○ yes
- ○ no/unsure

Have they, or anyone living with them, ever tested positive for **Coronavirus**?

- yes
- no/unsure

Have they, or anyone living with them, been hospitalized for a **new** respiratory illness since March 15th, 2020?

- yes
- no/unsure

Thank you for telling us about your fourth person!

Next, a few questions about the **fifth** and final person on your list!

block:p5

Now please consider the **fifth** and final person on the list.

Are they your sibling, parent, or child?

- yes
- no

What is their relationship to you?

- sibling
- parent
- child

What type of sibling?

- step
- half sibling; same mother
- half sibling; same father
- full sibling; same mother and father
- twin; identical
- twin; not identical

What type of parent?

- step
- biological

What type of child?

- step
- biological

What is their age? (In years)

What is their gender?

- ◯ woman
- ◯ man
- ◯ non-binary
- ◯ other

Are they left or right handed?

- ◯ left
- ◯ right
- ◯ neither/unsure

Where do you usually see them in person? (Please check all that apply)

- ☐ your current residence
- ☐ work
- ☐ commute
- ☐ social events

Have you seen them in person in the last 2 weeks?

- ◯ yes
- ◯ no

Have they developed any of these symptoms in the last 2 weeks? (Please check all that apply)

- ☐ fever above 101F for multiple days
- ☐ sudden loss of smell
- ☐ persistent and unusual head and body aches
- ☐ unexplained severe allergy symptoms
- ☐ respiratory symptoms requiring medical attention

Including them, about how many people share their living space?

How many of those are younger than 19 years?

How many of those are older than 64 years?

Have they, or anyone living with them, tested positive for the **Flu** since January 1st, 2020?

- ○ yes
- ○ no/unsure

Have they, or anyone living with they, ever tested positive for **Coronavirus**?

- yes
- no/unsure

Have they, or anyone living with them, been hospitalized for a **new** respiratory illness since March 15th, 2020?

- yes
- no/unsure

Thank you so much for helping us understand how Coronavirus and other respiratory illnesses move through our social lives.

To finish and submit the survey, click the Next button and then the Submit button on the next page.

Thank you again for your time!

Powered by Qualtrics
